# High performance bio-supercapacitor electrodes composed of graphitized hemicellulose porous carbon spheres

**DOI:** 10.3389/fbioe.2022.1030944

**Published:** 2022-09-29

**Authors:** Zhili Zhang, Fengfeng Li, Jiachuan Chen, Guihua Yang, Xingxiang Ji, Zhongjian Tian, Baobin Wang, Lei Zhang, Lucian Lucia

**Affiliations:** ^1^ State Key Laboratory of Biobased Material and Green Papermaking, Qilu University of Technology (Shandong Academy of Sciences), Jinan, China; ^2^ Department of Forest Biomaterials, North Carolina State University, Raleigh, NC, United States; ^3^ Department of Chemistry, North Carolina State University, Raleigh, NC, United States

**Keywords:** hemicelluloses, template-free, graphitic porous carbon spheres, electrode materials, supercapacitors

## Abstract

A template-free and one-step carbonization process was developed for fabricating graphitic porous carbon spheres (GPCSs) on hemicelluloses as the electrode material for supercapacitors. This method is green, low-energy, and less time consuming compared to the conventional two-step process (pore-forming and graphitizing). It uses K_2_FeO_4_, a mild activating agent that fulfills synchronous activation and graphitization. The GPCSs is regular spherical shape, have high nanoporosity, a large specific surface area (1,250 m2 g^−1^), and have a high graphitization degree. A unique structural advantage includes a rich interconnected conductive network for electron transfer that shortens the ion transport distance of the electrolyte. Remarkably, the GPCSs electrode displays outstanding electrochemical performance including high specific capacitance (262 F g^−1^ at 1.0 A g^−1^), rate capability energy (80%, 20 A g^−1^), and excellent cycling stability (95%, 10,000 cycles). This work represents a powerful methodology to develop sustainable and low-cost energy storage devices from hemicellulose.

## 1 Introduction

In recent years, environmental pollution and the shortage of petroleum-based resources have triggered an awareness worldwide among the people to study new energy devices replacing internal combustion engines. Supercapacitors are advanced energy storage devices that have attracted considerable research due to their good recyclability, rapid charging/discharging rate, high power density, low-cost, high life cycle, and environmental-friendly properties (Palchoudhury et al., 2019; Sun et al., 2021; Chambers et al., 2022; Yoshizawa-Fujita et al., 2022). The electrode materials are the important component governing their performance. Therefore, research on electrode materials has been a hot topic (Walters et al., 2021; Yu et al., 2021). Currently, porous carbon spheres are excellent candidates for electrode materials due to their physical morphology, chemical resistance, and well-developed porous structures (Veerakumar et al., 2020; Song et al., 2021; Liu et al., 2022; Yu et al., 2022). The regular spherical shape reduces the resistance of electrolyte diffusion, and the space between the spheres makes the electrode accessible to the electrolyte (Chen et al., 2014; Oschatz et al., 2015; Mutuma et al., 2019). Furthermore, the interconnected porous structure having a large specific surface area provides an ion-buffering reservoir, which can effectively shorten ion transport distance into the electrochemical active surface and greatly improve the capacitive performance (Du et al., 2010; Zhang et al., 2014). As reported, various chemical feedstocks such as pitch, coal, phenolic resin, styrene, and acetylene have been employed as conventional precursors to prepare porous carbon spheres through thermal condensation, arc discharge, chemical vapour deposition (CVD) and templating (Lee et al., 2006; Zhai et al., 2011; Ding et al., 2020). Among the various synthesis pathways for the fabrication of PCSs, templating is superior to other techniques because it leads to porous and regular spherical structures derived from hard or soft sacrificial templates (Doustkhah et al., 2021). However, the template method is time-consuming, costly, and complicated that involves template synthesis, infiltration, crosslinking and carbonization, template removal *and so on*, which largely limits commercial application. Therefore, from the perspective of sustainability, economics, and environment friendliness, there is an urgent need to develop a green, economical, efficient and sustainable porous carbon spheres displaying excellent capacitive properties.

Recently, a growing body of research has focused on cheap and renewable biomass resources as supercapacitive electrolytic feedstocks (Vijayakumar et al., 2019; Zhang et al., 2019; Xu et al., 2020; Peng et al., 2022). A multitude of low molecular weight carbohydrates such as lignin, chitosan, glucose, fructose, sucrose, xylose, have been explored to produce PCSs by mild hydrothermolysis (Saha et al., 2014; Xu et al., 2018; Gao et al., 2021; Hwang et al., 2022; Yu et al., 2022). But a paucity of studies has reported preparation of porous carbon spheres directly from high molecular weight carbohydrates such as cellulose and hemicelluloses (Wang and Shi, 2015; Hwang et al., 2022). Hemicellulose is a highly hydrophilic homo or heteropolysaccharide with a branched structure. It is one of the three major components found in the cell walls of lignocellulosic biomass, including glucose, galactose, mannose, xylose and others (Jha et al., 2019; Zhu et al., 2020). Owing to its abundance, good hydrophilicity and easy degradation, hemicelluloses are regarded as an ideal carbon precursor for the preparation of porous carbon spheres by hydrothermal carbonization. For electrode materials, they should not only have a large specific surface area and porous structure, but also have enough electrical conductivity to allow electron shuttling (Tan, et al., 2018; Tan, et al., 2021; Mohamed, et al., 2022). Currently, most reports use KOH or ZnCl_2_ as activators to improve the specific surface area of the bio-based hydrothermalized carbon spheres. Although the activated carbon spheres have a large specific surface area, the internal C skeleton is mostly amorphous carbon, which leads to a decrease in the conductivity. At present, there are a few reports on the preparation of porous carbon spheres by hydrothermolysis of hemicelluloses and associated hydrolysates, but obtained samples still have defects such as low graphitization degree or serious structural damage, which will greatly diminish the capacitance performance for electrode materials.

Herein, a template-free and one-step carbonization process was developed for fabricating GPCSs based on hemicelluloses through a hydrothermal method. During the carbonization, K_2_FeO_4_ was used as both activator and catalyst to complete the activation and graphitization simultaneously. Compared with conventional two-step carbonization, this way is simple, time-saving and mild, which reduces excessive damage to the morphology of the as-produced carbon spheres. Therefore, the obtained GPCSs has regular spherical morphology, a 3D porous network structure, and high graphitization. In addition, a bio-supercapacitor is rationally designed by symmetric GPCSs electrodes. Such a GPCSs based supercapacitor shows higher specific performance and cycle stability.

## 2 Materials and methods

### 2.1 Materials

Hemicelluloses with weight-average molecular weight (Mn) of 9,300 g/mol and number-average molecular weight (Mw) of 14,350 g/mol, was obtained from a mill in Xinjiang Province, China. The content of glucose, xylose, galactose, and arabinose were, respectively, 83.38%, 2.59%, 0.16%, and 0.16%. K_2_FeO_4_, KOH, HCl and other reagents were purchased from Beijing Chemical Reagent Co., China. All chemicals used in this study were of analytical grade used without any further purification.

### 2.2 Synthesis of graphitic porous carbon spheres

**FIGURE 1:**
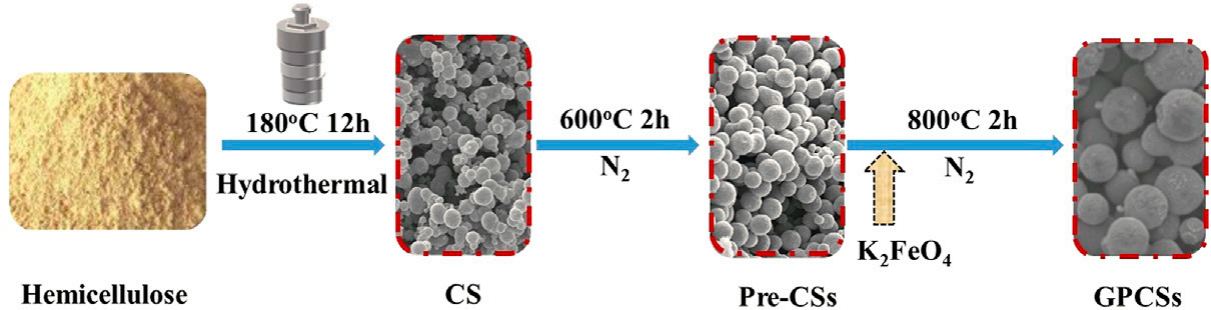
The synthetic route of GPCSs.

The GPCSs were prepared as follows (Figure 1): 2.5 g hemicelluloses and 25 ml deionized water were stirred at room temperature for 15 min to form a stable suspension and added to a 40 ml Teflon autoclave. Subsequently, the mixed solution was maintained at 180°C for 12 h. After cooling, solid samples were separated by vacuum filtration, cleaned several times with water and ethanol, and dried in a vacuum drying oven at 80°C overnight to obtain hydrothermalized carbon spheres (CSs). Secondly, the carbon spheres (CSs) were pre-carbonized at 600°C for 2 h at a heating rate of 5°C min^−1^ under an N_2_ atmosphere. The samples obtained were labeled as pre-carbon spheres (Pre-CSs). Finally, the Pre-CSs were mixed with K_2_FeO_4_ or KOH in 30 ml deionized water at a mass ratio of 1:3 with continuous stirring overnight and dried in a vacuum drying oven at 80°C for 8 h obtained as two dry solid samples. The above solids were activated in the tube furnace at 800°C for 2 h. After reaction, the resulting products were washed successively with 1 M HCl solution and deionized water, and then dried at 80°C. The obtained samples were denoted as GPCSs-1, GPCSs-2, respectively. The final yield of GPCSs is approximately 35%. The control group was treated with water alone in a volume commensurate to the aqueous K_2_FeO_4_ or KOH solution (denoted as GPCSs-0).

### 2.3 Characterizations

The surface morphology of dried samples was analyzed by scanning electron microscopy (SEM, Hitachi S-5500). The samples were spread on a circular base with highly conductive double-sided tape and covered with a thin layer of sputtered gold by magnetron sputtering equipment. The functional groups of samples were analyzed by a Fourier-transform Infrared Ray (FT-IR) spectrometer (VERTEX 70, Bruker, German) from 500–4,000 cm^−1^ at resolution of 0.5 cm^−1^. Raman patterns were recorded by Raman spectroscopy (Bruker Optics, German) equipped with a 532 nm laser beam. X-ray diffraction (XRD) patterns of samples were carried out on a Bruker Diffractometer with Cu-Kα radiation (Bruker, D8 ADVANCE), and data were collected from 2θ = 5–60°. ASAP 2460 to observe the nitrogen adsorption isotherms at −196°C. Moreover, the specific surface area and pore size distribution were evaluated from the adsorption branch of the isotherm using Brunauer–Emmett–Teller theory (BET) and Barrett–Joyner–Halenda model (BJH). CE-440 Analytics (EAI) was employed to test the elemental mappings. X-ray photoelectron spectroscopy (XPS) spectra was performed by an Axis Ultra DLD spectrometer (UKESCALAB 250Xi, USA) with monochrome Al Kα radiation (hv = 486.6 eV). Transmission electron microscopy (TEM) were analyzed by a field emission Tecnai G2 F20 electron (Hillsboro, OR, United States) microscope. The morphologies of the samples were observed by a transmission electron microscope (JEM-2100, JEOL, Japan).

The carbon material samples, carbon black (CB) and polytetrafluoroethylene (PTFE) solution were mixed in the weight ratio of 8:1:1. Then, the mixture was stirred and ground at room temperature until a uniform slurry was obtained. The uniform slurry was coated on the Pt current collector at a size of 1 cm*2 cm and dried at ambient temperature for 24 h. A single electrode was obtained. Approximately 3 mg of active carbon materials were loaded in each electrode.

The two-electrode configuration for studying the electrochemical properties of the samples was a CHI760e electrochemical workstation (Shanghai Chen Hua Instruments Co., China). The cyclic voltammetry (CV) curves were operated over a voltage window of 0–1 V with a voltage sweep rates of 5–200 mV s^−1^. Electrochemical impedance spectroscopy (EIS) measurements were recorded in the frequency range from 0.01 Hz to 100 kHz at an open circuit potential with an amplitude of 10 mV. The galvanostatic charge-discharge tests (GC) were employed to measure the cycling stability at a constant current density of 10 A g^−1^ for 10,000 cycles. The specific capacitance (C_m_) was calculated according to Eq. 1:

$$C_{m}=\frac{2I_{d}}{\frac{∆V}{∆t}\times m}$$ (1)

where C_m_ (F g^−1^) is the specific capacitance, I_d_ (mA) is the discharge current, ∆V (V) is discharged voltage range, ∆t (s) is the discharge time, and m (g) is the mass loading of the active material on a single electrode.

The energy density (E, Wh kg^−1^) and power density (P, W kg^−1^) of the carbon materials based device were calculated according to Eqs 2, 3 (Zhou et al., 2018):

$$E=\frac{C_{m}∆V^{2}}{8\times 3.6}$$ (2)

$$P=3600\times \frac{E}{∆t}$$ (3)

where C_m_ (F g^−1^), ΔV (V), and Δt (s) are the specific capacitance based on the two electrodes, the discharge voltage range exclusive of the IR drop, and the discharge time, respectively.

## 3 Results and discussion

### 3.1 Morphology and structure of CSs and Pre-CSs

Typical SEM images of CSs and Pre-CSs are shown in Supplementary Figure S1. CSs has a regular spherical structure with a smooth surface and a size distribution ranging from 0.5 to 2 μm in diameter (Supplementary Figures S1A,C). However, there are irregular flaky products, and spheres connected that appear to be irregular flaky products, several of which are clustered. It is worth noting that the Pre-CSs shows a more regular spherical structure with good dispersion and rough surfaces (Supplementary Figures S1B,D).

**FIGURE 2:**
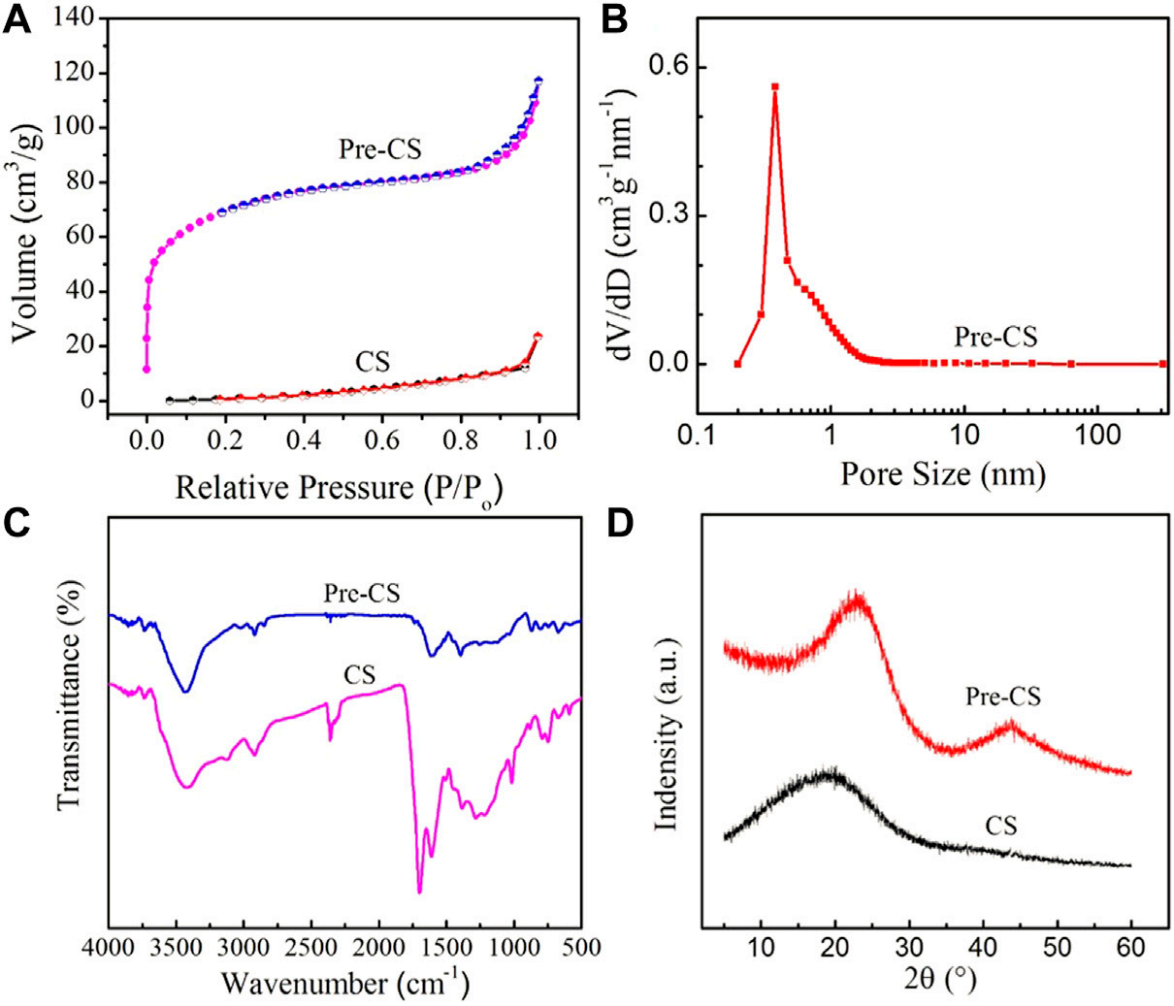
**(A)** Nitrogen adsorption-desorption isotherm of Pre-CSs and CSs. **(B)** Pore size distribution of Pre-CSs. **(C)** FTIR spectra of Pre-CSs and CSs. **(D)** XRD patterns of Pre-CSs and CSs.

To further verify the microstructure change, carbon spheres before and after pre-carbonization were analyzed by measuring the pore size distribution and N_2_ adsorption-desorption (Figures 2A,B). The specific surface area of CSs is 17 m2 g^−1^, whereas Pre-CSs possess 215.2 m2 g^−1^, which is consistent with the above SEM results. In this pre-carbonization process, the increase of specific surface area is mainly due to the condensation polymerization of oxygen-containing functional groups on the carbon spheres, which generates many small molecules (such as CO_2_ and H_2_O). As these small molecules vaporize, a part of micropores is formed, which increases the specific surface area. In addition, the formation of pores is conducive to the diffusion of the activator into the sphere during the subsequent activation process, thus reducing the concentration of the activator on the surface of the sphere to ensure the spherical shape of the carbon spheres.

To study the surface chemistry of CSs and Pre-CSs, qualitative identification of the functional groups by FTIR was undertaken over the range of 4,000–400 cm^−1^ (Figure 2C). These two carbon spheres presented typical band information such as -OH (3,400 cm^−1^), C-OH (1,020–1,380 cm^−1^) and C=C plane stretching vibration of benzene ring (1,610 cm^−1^), which indicates that both samples contain oxygen functional groups and aromatic rings. Notably, the C-O (1,261 cm^−1^) and O-C=O (1702 cm^−1^) peak intensities of Pre-CSs decrease significantly. This confirms that the oxygen functional groups are condensed into small molecules and then removed in the pre-carbonization process. The decrease of oxygen functional groups was also supported by the result of elemental analysis (Table 1). As compared with the CSs, the oxygen content of Pre-CSs decreased from 25.43% to 4.025% consistent with previous data.

**TABLE 1:** Elemental analysis of hemicelluloses-based carbon spheres.

Samples ID	C/%	H/%	O/%	N/%
CSs	70.52	4.013	25.43	0.041
Pre-CSs	93.56	2.390	4.025	0.025
GPCSs-0	96.10	0.934	2.821	0.145
GPCSs-1	86.00	0.569	11.674	1.757
GPCSs-2	83.12	0.331	16.321	0.228

The X-ray diffraction (XRD) pattern was carried out to further investigate the crystal structure of carbon spheres before and after pre-carbonization. As shown in Figure 2D, the CSs only has a very wide diffraction peak at ∼2θ = 19.8°, corresponding to the (002) crystal plane of graphite microcrystalline. Unlike CSs, the (002) diffraction peak of the Pre-CSs was slightly narrow and shifted right to 2θ = 22.5°, closer to the peak position of intact graphite (2θ = 26.5°). Moreover, a second diffraction peak was appeared at 2θ = 43.8°, corresponding to the (100) crystal plane of the graphite microcrystalline (JCPDS Card No.99-0057). The results show that pre-carbonization process can significantly increase the graphitization degree of hydrothermal carbon spheres, which can reduce the surface activation rate during the activation process to maintain the intact spherical structure. Therefore, according to results of SEM, FTIR, XRD, and composition characterization, Pre-CSs has a spherical shape, partially microporous and graphitized structure, an ideal precursor for subsequent activation experiments.

### 3.2 Characterization of graphitic porous carbon spheres

#### 3.2.1 Morphological of graphitic porous carbon spheres

For the electrode material, it should not only have a large specific surface area and porous structure but have sufficient electrical conductivity to complete electron transmission. Most of the biomass-based porous carbon spheres reported used KOH, H_3_PO_4_ and ZnCl_2_ as activators to improve the porous structure of hydrothermalized carbon spheres to increase their specific surface areas. Although the activated carbon spheres have a large specific surface area, most of the C in the internal skeleton is amorphous carbon leading to reduced conductivity of the porous materials, an attractive defect for electrode materials (Jain et al., 2016). Therefore, K_2_FeO_4_ was used as both activator and catalyst to complete activation and graphitization simultaneously for the preparation of hemicelluloses-based GPCSs.

**FIGURE 3:**
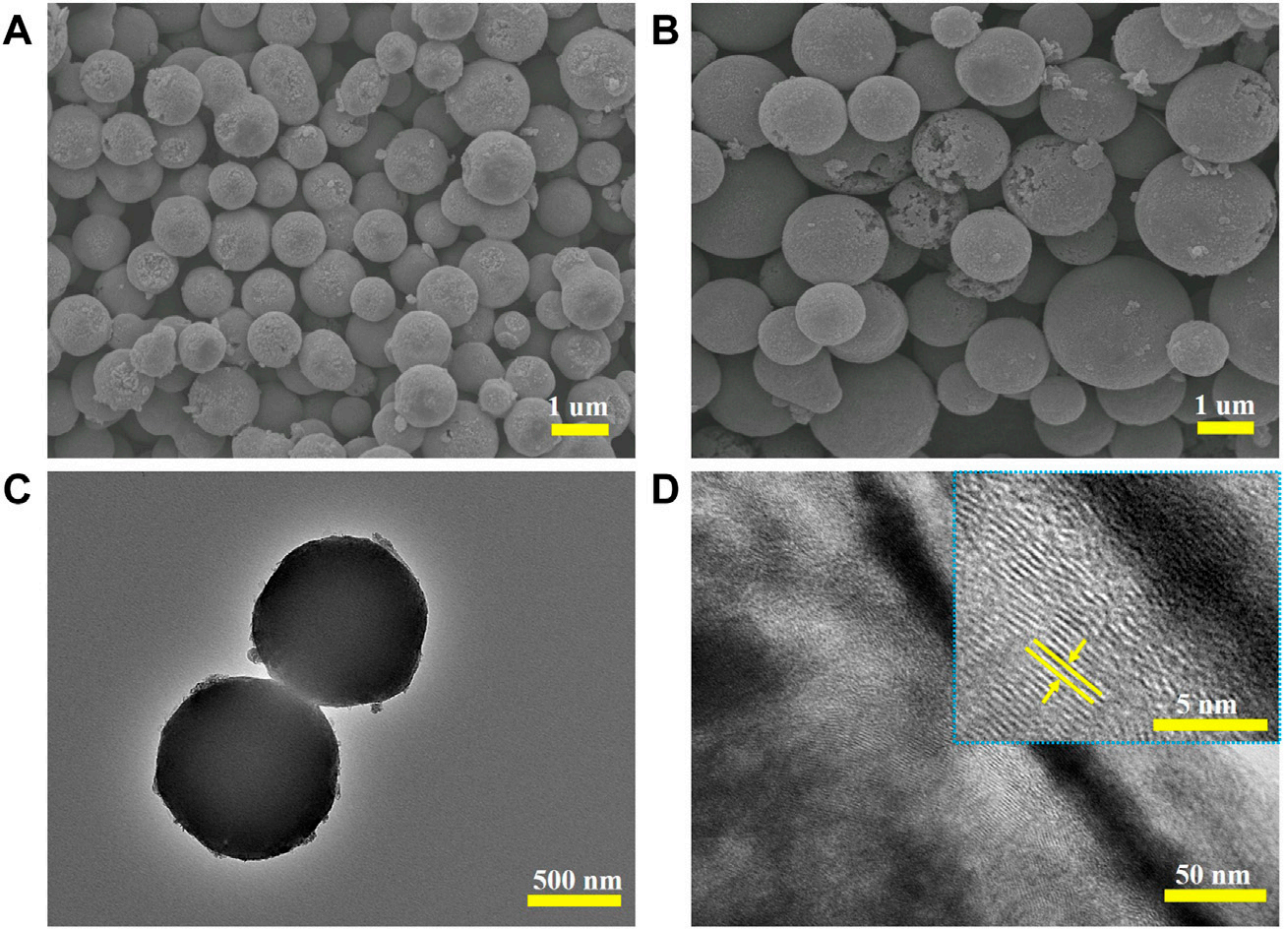
**(A)** GPCSs-1, and **(B)** GPCSs-2. **(C)** TEM and **(D)** high-resolution TEM images of GPCSs-1.

The microtopography of the samples activated by different activators was characterized by SEM. In Figure 3 and Supplementary Figure S2, the samples without activator (GPCSs-0), K_2_FeO_4_ activation (GPCSs-1) and KOH activation (GPCSs-2) all give rise to spherical shapes. However, by further surface micromorphology comparison, spherical surfaces of GPCSs-1 and GPCSs-2 (Figures 3A,C) were very rough and exhibited obvious etching marks while the surface of GPCSs-0 (Supplementary Figure S2) was relatively smooth indicating that K_2_FeO_4_ and KOH play an important role in pore-forming. The spherical shape ensures full contact between electrode materials and electrolyte, while the rough surface is conducive to penetration of the electrolyte and provides more electrochemically active sites for charge storage. To further investigate the graphitized microstructure of the GPCSs-1, TEM and high resolution. TEM images were taken on the activated sample with K_2_FeO_4_. As shown in Figure 3C, the TEM image of GPCSs-1 demonstrated its regular uniform spherical morphology. Furthermore, the continuous porous structure could be found in the high-resolution TEM image (Figure 3D). In addition, the inset showed ordered lattice fringes with a distance of 0.338 nm, corresponding to the graphite (002) plane. It demonstrates that GPCSs-1 has great degree of graphitization, which should cause high electric conductivity of the sample.

#### 3.2.2 Structural characterization of graphitic porous carbon spheres

**FIGURE 4:**
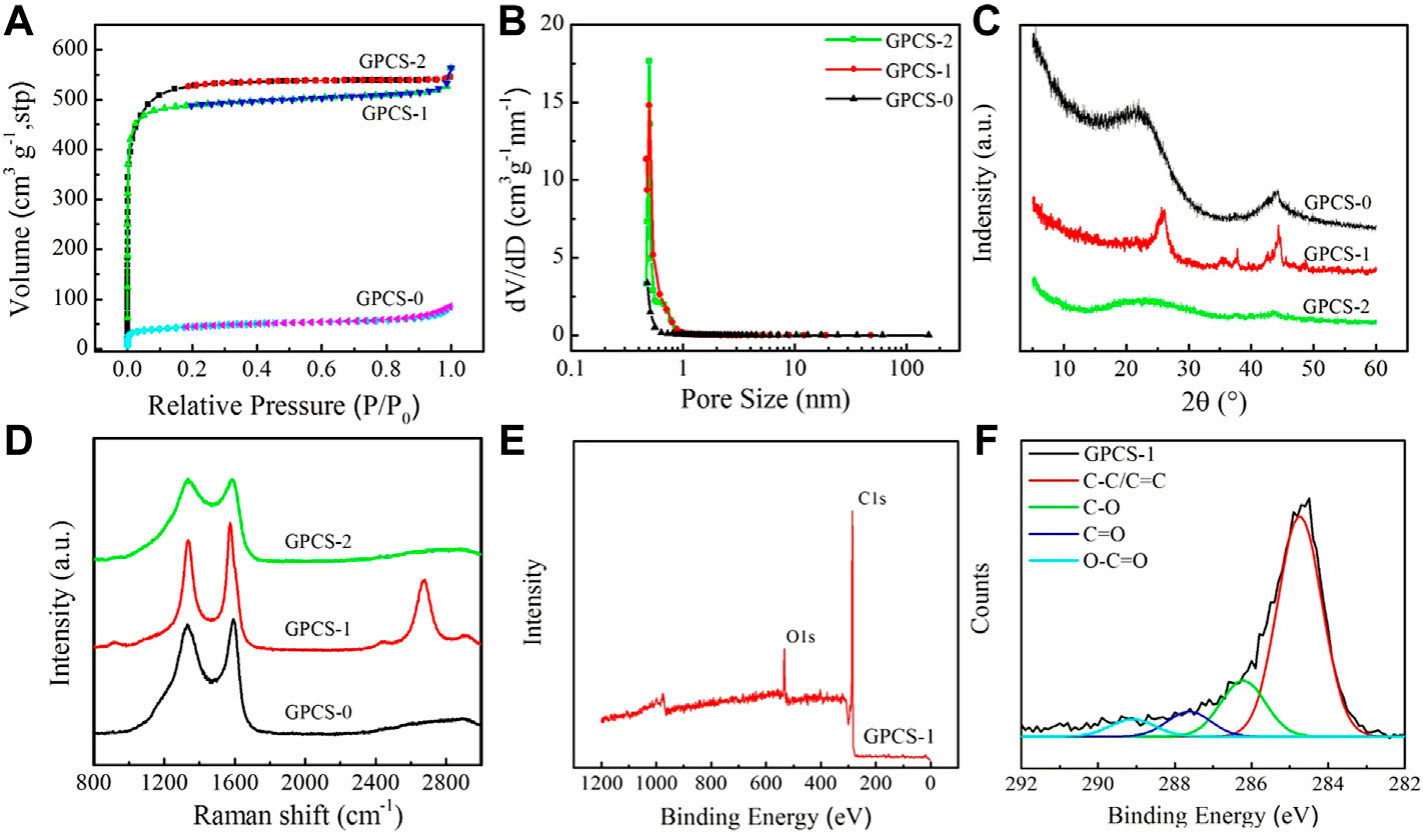
**(A)** Nitrogen adsorption-desorption isotherm. **(B)** Pore size distribution of GPCSs-0, GPCSs-1 and GPCSs-2. **(C)** XRD patterns and **(D)** Raman spectra of GPCSs-0, GPCSs-1 and GPCSs-2. **(E)** XPS survey spectra and **(F)** High-resolution C1s peaks spectra of for GPCSs-1.

To further understand the changes of specific surface area and pore size distribution for activated carbon spheres, N_2_ adsorption and desorption tests were carried out on three samples GPCSs-0, GPCSs-1, and GPCSs-2. In Figure 4A, both GPCSs-1 and GPCSs-2 provided relatively high BET specific surface areas (1,250 and 1,440 m2 g^−1^, respectively). Their adsorption and desorption isotherms exhibit typical IUPAC-I curves pointing to the existence of many micropores. In Figure 4B, the pore size of GPCSs-1 and GPCSs-2 is between 0.5–1 nm, which confirms the above results. Studies have shown that the smaller the micropore size of the electrode material, the greater the charge storage. However, for electrolytes in water, hydrated ions cannot enter pores less than 1 nm which cannot lead to charge storage (Inagaki et al., 2009; Luo et al., 2021). A reasonable distribution of the nonporous size of GPCSs-1 and GPCSs-2 provides favorable conditions for electrolyte ion storage. As compared to the two activated samples, the specific surface area of GPCSs-0 without activator is only 275 m2 g^−1^. Moreover, it did not significantly increase compared with the precursor sample of Pre-CSs (215.2 m2 g^−1^). By applying only high temperature treatment, there is a simple carbonization process that further removes oxygen functional groups and increases the proportion of C (Table 1). However, it has no obvious effect on pore formation. The N_2_ adsorption-desorption test results are consistent with SEM, both of which indicated that GPCSs-1 and GPCSs-2 possess large specific surface areas and good pore structures, prerequisites for electrode materials.

Similarly, the conductivity of the electrode materials is also one of the key factors to determine electrochemical performance. The conductivity of electrode materials can be visually shown by graphitization degree. The graphitization degree of samples was obtained under different treatment conditions and monitored by XRD and Raman. As shown in Figure 4C, GPCSs-0 has two broad diffraction peaks at 23.3° and 43.8°, respectively corresponding to the (002) and (101) crystal plane reflections of the graphite lattice indicating that the carbonized sample at 800 °C has a certain degree of graphitization. However, after KOH activation, both diffraction peaks of GPCSs-2 were significantly attenuated, mainly because KOH activation damaged the order degree of biomass carbon to a certain extent, resulting in decrease of graphitization degree. Notably, when K_2_FeO_4_ was used as the activator, the XRD curve of the GPCSs-1 indicated that the reflection peaks of the (002) and (101) crystal planes representing the graphite carbon became sharp and shifted to 26.3° and 44.2° (JCPDS Card No.99-0057). This means that the K_2_FeO_4_ activation did not destroy the order of the carbon sphere structure, but promoted the formation of a graphitized structure, thereby increasing electrical conductivity.

Raman spectra also further proved the difference of graphitization structure of different samples. In Figure 4D, two strong peaks, corresponding to D (∼1,350 cm^−1^) and G (∼1,580 cm^−1^) band, can be observed in the Raman spectra of all samples. The G band is related to the graphitic order, while the D band is referred to the disordered and imperfect structures in the carbonaceous materials. The integral intensity ratio (I_D_/I_G_) is used to estimate to indicate the level of graphitic ordering in the carbon materials. For the GPCSs-0, the I_D_/I_G_ ratio of the carbon spheres without activator is ∼0.95. After KOH activation, the D and G band of GPCSs-2 sample became broader, presenting a superposition trend. Moreover, the I_D_/I_G_ ratio increased to 1.02. These results indicate that KOH activation increases specific surface area of the carbon spheres and increases defects of graphite microcrystals resulting in an increase in disorder and a decrease in graphitization. Obviously, after activation by K_2_FeO_4_, the D and G peaks were sharp, and the I_D_/I_G_ ratio also decreased to 0.83, which proved that the high percentage of ordered carbon exists in the GPCSs-1 samples. It is worth noting that the spectrum of GPCSs-1 not only displayed D and G bands, but also displayed a sharp peak at about 2,700 cm^−1^, which is the 2D band of graphene. This means that there is a very high graphene carbon content in GPCSs-1. The Raman spectroscopy results confirmed that K_2_FeO_4_ activation could not only increase the specific surface area (1,250 m^2^ g−1), but could also greatly improve the graphitization degree of carbon spheres, consistent with the XRD results shown in Figure 4C.

The surface chemistry of GPCSs-1 activated with K_2_FeO_4_ was further studied by XPS (Figures 4E,F). Two dominant peaks appeared at 531.5 and 284.8 eV in the XPS survey spectra, respectively, belonging to O1s and C1s, which directly confirmed the presence of oxygen and carbon in the samples. The atomic C and O content were calculated to be 87.29% and 9.71%, respectively (Figure 4E). The existence of O element (11.74%) was also supported by the result of elemental analysis (Table 1). As shown in Figure 4F, the high-resolution spectrum of C 1s of GPCSs-1 could be divided into three peaks, corresponding to C-O (286.2 eV), C=O (287.8 eV) and O-C=O (289.2 eV), respectively. The presence of a small amount of oxygen-containing functional groups could increase the infiltration of electrolyte on the surface of the electrode materials, to reduce the transport resistance of the electrolyte conducive to improvement of electrochemical performance. Thus, according to the above results, GPCSs-1 has the most ideal specific surface area, pore size distribution, graphitization degree (electro-conductivity) and hydrophilic groups, expected to be an ideal electrode material with high electrochemical performance for supercapacitors.

#### 3.2.3 Activation mechanism of K_2_FeO_4_

The activation mechanism of K_2_FeO_4_ may now be explained. First, K_2_FeO_4_ is decomposed into KOH and Fe(OH)_3_ in a slightly acidic system, which play the roles of activation and catalysis, respectively. The role of KOH is mainly pore-forming. After a variety of physical and chemical activations, the carbon lattices expanded irreversibly and resulted in high specific surface area and hierarchical porous. Moreover, in the process of K_2_FeO_4_ reduction to Fe(OH)_3_, the iron ions undergo a series of intermediate states from Fe^6+^ to Fe^3+^, during which the iron ions with positive charges can be stably adsorbed on the surface of carbon spheres rich in oxygen-containing functional groups. This ensures uniform distribution on the surface of the sample to be activated. Second, in the process of high temperature treatment, Fe(OH)_3_ is gradually reduced to metallic iron which finally behaved as a catalyst for the conversion of amorphous carbon into graphitic carbon thus leading to enhanced graphitization degree (Hoekstra et al., 2016; Gong et al., 2017), as described by Eqs 4–6.

$$Fe{\left(OH\right)}_{3}\to FeO\left(OH\right)\to {Fe}_{2}O_{3}$$ (4)

$${3Fe}_{2}O_{3}+\left(H_{2},C,CO\right)\to {2Fe}_{3}O_{4}+\left(H_{2}{O,CO,CO}_{2}\right)$$ (5)

$${Fe}_{3}O_{4}+4\left(H_{2},C,CO\right)\to 3Fe+4\left(H_{2}{O,CO,CO}_{2}\right)$$ (6)

Therefore, after K_2_FeO_4_ activation, the obtained samples have regular spherical shape, large specific surface area and hierarchical porosity, and high graphitization degree, i.e., high electrical conductivity. All these structural advantages indicate that GPCSs-1 has the potential to be used as highly efficient electrode material for supercapacitors.

### 3.3 Electrochemical properties of graphitic porous carbon spheres

**FIGURE 5:**
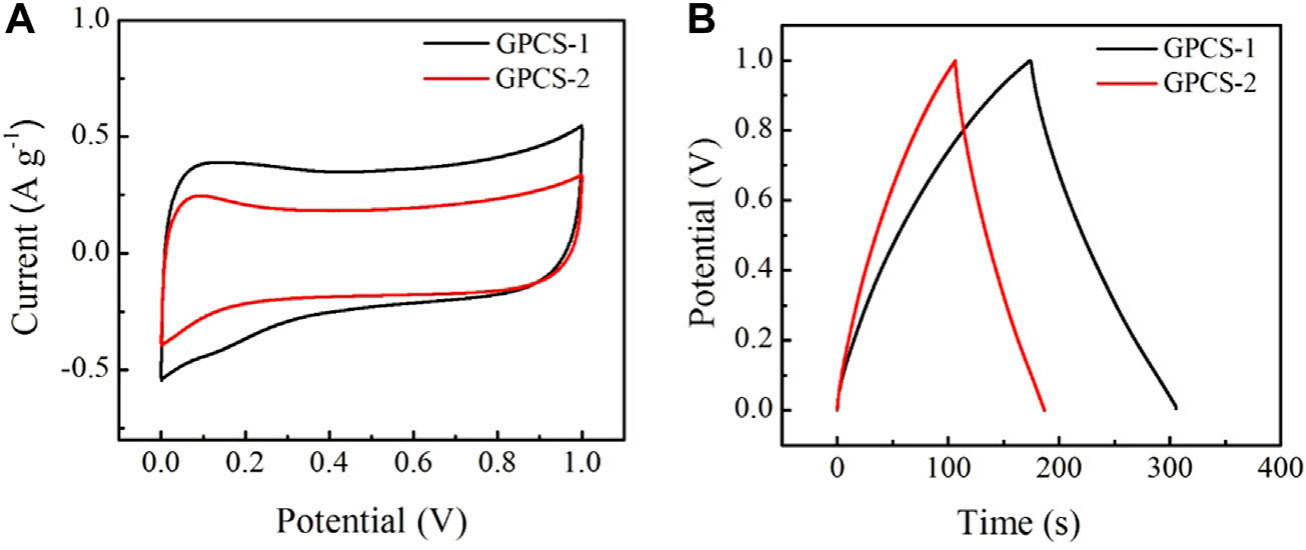
Capacitive performances of GPCSs-1 and GPCSs-2 symmetrical supercapacitors in 1 M H_2_SO_4_ aqueous electrolyte. **(A)** CV curves at a scan rate of 5 mV s^−1^. **(B)** GCD curves at a current density of 1 A g^−1^.

To investigate the electrochemical performance, GPCSs-1 was used as electrodes for assembling symmetric supercapacitors in a 1 M H_2_SO_4_ aqueous electrolyte. The supercapacitors have a three-layer structure: electrode-electrolyte-electrode with a final thickness of 2 mm, diameter of 2.0 cm. For comparison, a GPCSs-2 electrode-based symmetric device was also fabricated using the same method. The cyclic voltammetry (CV) measurements were employed to determine the capacitive performances. As depicted in Figure 5A, both CV curves of GPCSs-1 and GPCSs-2 electrodes at 5 mV s^−1^ presented a representative quasi-rectangular shape, indicating that both GPCSs-1 and GPCSs-2 electrodes have an ideal electrical double-layer capacitance. The area in the CV curve for GPCSs-1 is apparently much higher than those of the GPCSs-2 electrode at the same scan rate. As expected, the capacitor storage capacity of the GPCSs-1 electrode is better than that of GPCSs-2.

To further evaluate the electrochemical performance, galvanostatic charge/discharge cycling (GCD) was used to calculate the specific capacitances. As displayed in Figure 5B, the GCD curves of GPCSs-1and GPCSs-2 electrodes at 1.0 A g^−1^ showed quasi-symmetrical triangular shapes characteristic of the behavior of electric double-layer capacitance. As shown in the discharge-time curve, the discharge time of GPCSs-2 was less than that of GPCSs-1, which signifies that the specific capacitance of the GPCSs-2 electrode is smaller than the GPCSs-1 electrode. The specific capacitance of GPCSs-1 based supercapacitor calculated from GCD curves is 262 F g^−1^ at current densities of 1.0 A g^−1^. The performances are better than GPCS-2 based supercapacitor (169 F g^−1^) at the same current density. This result was consistent with CV measurements. According to the above analysis, one possible reason for this improvement is that the GPCSs-1 electrode has a high degree of graphitization, conducive to specific capacitance electrochemical performance. The mass-specific capacitance of GPCS-1 surpassed some commercial activated carbon, such as SPC-01 (190 F g^−1^), YEC-100 (80–100 F g^−1^).

**FIGURE 6:**
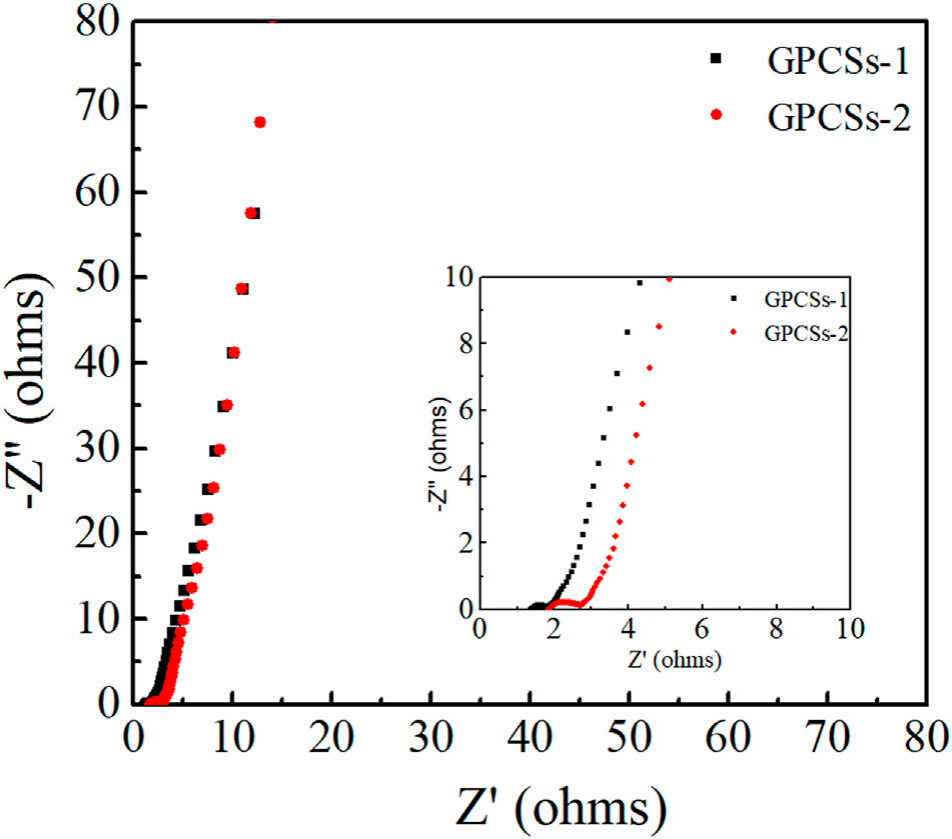
Nyquist plots of phase angle versus frequency.

Furthermore, the electrochemical impedance spectroscopy (ESI) was obtained to evaluate the ion-transport behavior and electrical resistances of electrodes. Nyquist plots of GPCSs-1 and GPCSs-2 electrodes at frequencies from 0.01 to 10^5^ Hz are given in Figure 6. The inset shown an enlarged view of the high-frequency region. In the low-frequency region, the Nyquist plots of two samples were nearly perpendicular to the Z” axis, which exhibited an ideal capacitive behavior. A significant difference in the AC impedance curves of the two samples occurred in the high frequency region, where the intercept of the real axis and the semicircle isolator represented the equivalent series resistance (R_es_) and the interface charge transfer resistance (R_ct_) of the capacitor, respectively. The R_es_ (1.3 Ω) and the R_ct_ (0.4 Ω) of GPCSs-1 was obviously smaller than that of GH (R_es_ 2.0 Ω, R_ct_ 0.8 Ω). The results indicating that the GPCSs-1 electrodes have high charge/ion transport efficiency due to the high degree of graphitization and conductivity. The analysis of CV and GCD curves, as well as EIS, all proved that the capacitance performance of GPCSs-1 electrode was obviously better than GPCSs-2 electrode.

**FIGURE 7:**
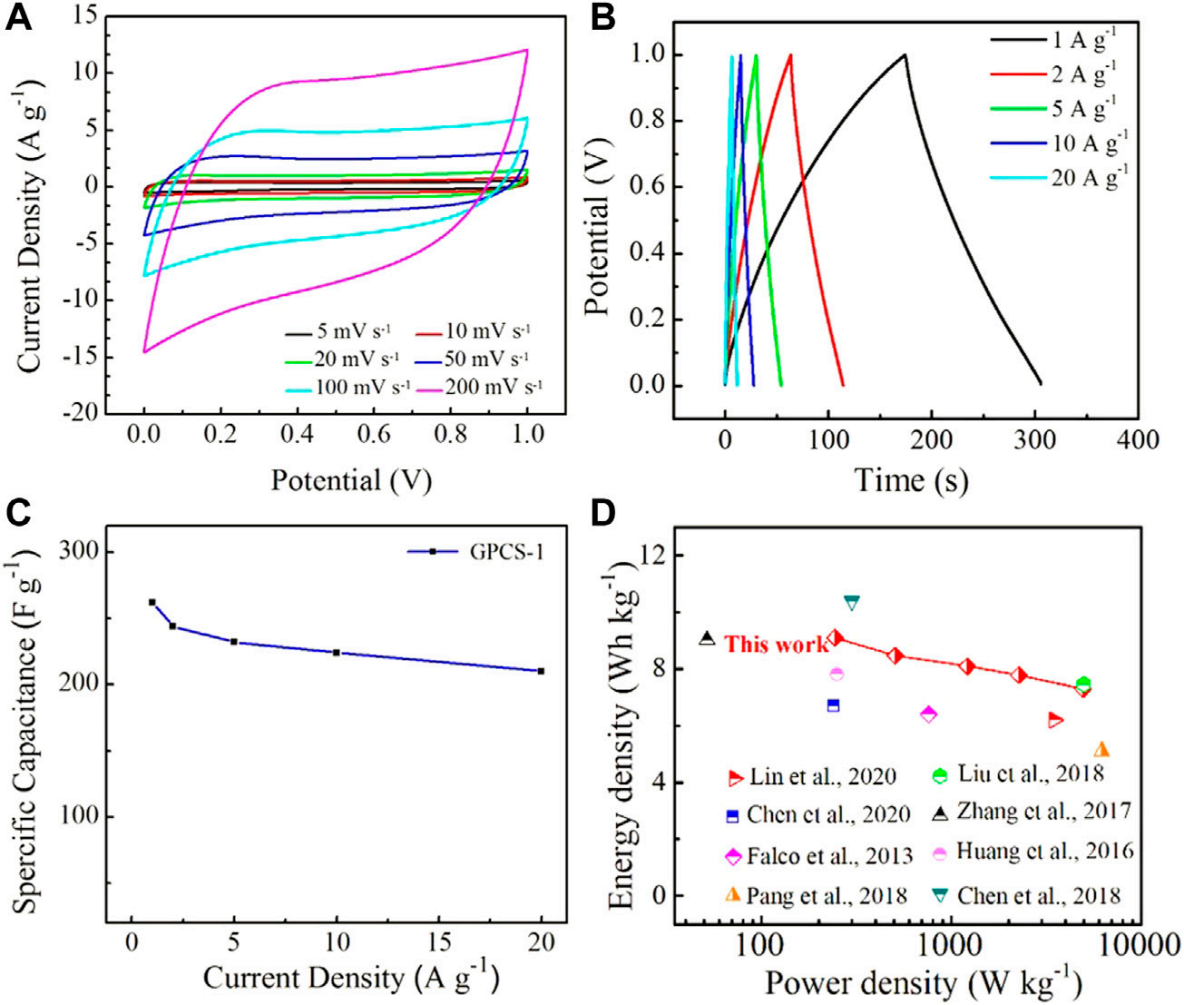
**(A)** CV curves of GPCSs-1 symmetric supercapacitor in 1 M H_2_SO_4_ aqueous electrolyte at various scan rates ranging from 5 to 200 mV s^−1^. **(B)** GCD curves of GPCSs-1 symmetric supercapacitor in 1 M H_2_SO_4_ aqueous electrolyte at different current densities ranging from 1 to 20 A g^−1^. **(C)** Specific capacitances versus different current densities. **(D)** Comparison of the Ragone plot of GPCSs with those of some representative carbon-based supercapacitors from biomass.

To further evaluate the electrochemical performance of the GPCSs-1 electrode-based symmetric supercapacitor, CV curves at various scan rates ranging from 5 to 200 mV s^−1^ and GC curves at different current densities ranging from 1 to 20 A g^−1^ were employed. As seen in Figure 7A, the CV curves at a scan rate of 200 mV s^−1^ still provided rectangular shapes, indicating that the charge could be efficiently and quickly transferred inside the device. In addition, at high current density, the GC curve of GPCSs-1 presented a symmetrical triangular shape with no obvious voltage drop (Figure 7B). As the current density increased to 20 A g^−1^, the specific capacitance reduced to 210 F g^−1^ with 80% of the initial capacitance retention, highlighting an excellent rate capability (Figure 7C). The excellent high-rate capability is mainly due to the multiple-porous channels in GPCSs-1, which could afford more efficient pathways for ion diffusion. Moreover, the GPCSs-1 electrode showed excellent electrochemical stability with 95% capacitance retention over 10,000 cycles at a high current density of 10 A g^−1^ (Supplementary Figure S3).

To further evaluate the overall performance of our devices, a Ragone plot was drafted (Figure 7D) (Falco et al., 2013; Huang et al., 2016; Zhang et al., 2017; Chen et al., 2018; Liu et al., 2018; Pang et al., 2018; Chen et al., 2020; Lin et al., 2020). In the aqueous electrolyte system, the GPCSs-1-based device exhibited a high energy density of 9.10 Wh kg^−1^ at a power density of 244.5 W kg^−1^, and still retained 7.29 Wh kg^−1^ at a power density of 4,951.7 W kg^−1^, which is mainly due to its excellent rate capability. In addition, the energy-power characteristics of the GPCSs-1-based device was compared with other previously reported carbon-based electrode supercapacitors (Table S1). The high energy density is comparable or superior to the supercapacitors based on advanced biomass porous carbon materials such as rice straw-derived porous carbon spheres (7.46 Wh kg^−1^, 5000 W kg^−1^), HTC carbon hollow spheres (6.4 Wh kg^−1^, 760 W kg^−1^), carbon spheres derived from sodium lignosulfonate (5.1 Wh kg^−1^, 6200 W kg^−1^), hemicelluloses-derived porous activated carbon (6.2 Wh kg^−1^, 3,498.8 W kg^−1^), activated carbon fibers (7.8 Wh kg^−1^, 250 W kg^−1^), *etc*., The accessing of good rate and cycling stability is mainly attributed to good conductivity, regular spherical shape, larger specific surface area, and multiple-porous structures from the GPCSs-1 electrode.

## 4 Conclusion

In summary, GPCSs with well-shaped, large specific surface area (1,250 m^2^ g^−1^) and high graphitization were successfully prepared from hemicelluloses via a green, low energy consumption, and efficient method. During the carbonization process, K_2_FeO_4_ was converted to KOH and Fe, which was utilized as the activating agent and catalyst, respectively, to realize the synchronous activation and graphitization of carbon spheres. Benefitting from the mild one-step carbonization method, the GPCSs-1 possessed an interconnected conductive network and multileveled porous structure. Therefore, the GPCSs-1 electrodes showed excellent electrochemical performances, such as high specific capacitance (262 F g^−1^ at 1.0 A g^−1^), high rate capability energy (20 A g^−1^, 80%) and cycling stability (95%, 10,000 cycles), demonstrating great potential as sustainable, low cost, and bio-based high-performance electrode materials for supercapacitors.

## Data Availability

The original contributions presented in the study are included in the article/Supplementary Material, further inquiries can be directed to the corresponding authors.
