# Supplementary material for: High performance bio-supercapacitor electrodes composed of graphitized hemicellulose porous carbon spheres
Source: Front Bioeng Biotechnol. 2022 Sep 29;10:1030944. doi: 10.3389/fbioe.2022.1030944 (PMC9556887; doi:10.3389/fbioe.2022.1030944)
Supplement: Supplementary file 1 [file DataSheet1.DOCX]

Supplementary information for

High performance bio-supercapacitor electrodes composed of graphitized hemicellulose porous carbon spheres

**Figure S1** SEM images of the (a, c) CSs and (b, d) Pre-CSs.

**Figure S2** SEM images of the (a) GPCSs-0


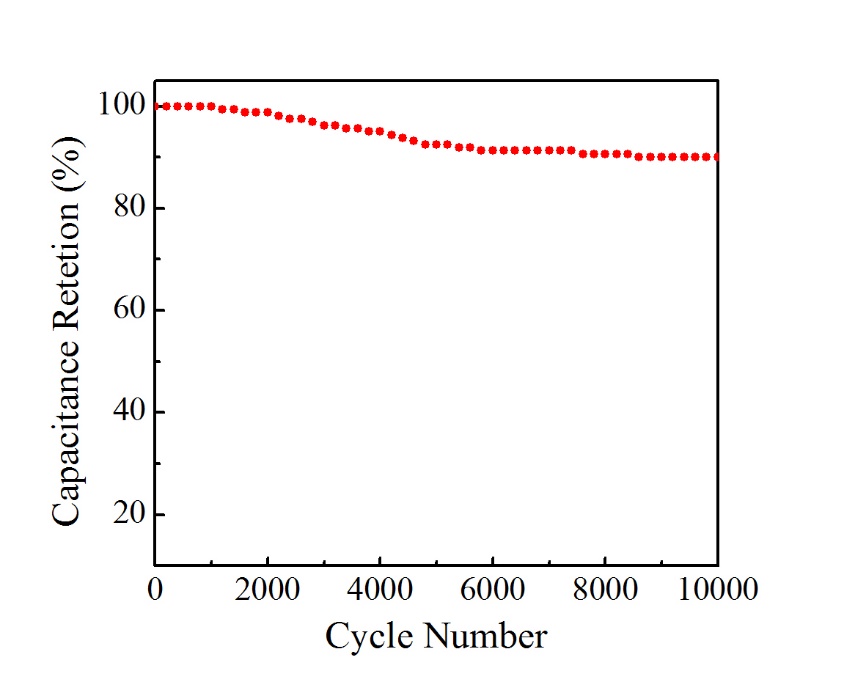


**Figure S3** Cycling stability of GPCSs-1 symmetric supercapacitor at a current density of 10 A/g.

**Table S1.** Comparison of various biomass-carbonaceous electrode based supercapacitors in an aqueous electrolyte system

| Materials | Electrolyte | Activating agent | Power density (W/kg) | Energy density  (Wh/kg) | Ref. |
| --- | --- | --- | --- | --- | --- |
| Heteropolysaccharide-derived porous activated carbon | 6M KOH | ZnCl_2_ | 3498.8 | 6.2 | Lin et al., 2020 |
| Rice husk-based hierarchical porous carbon | 6M KOH | NaOH | 241 | 6.7 | Chen et al., 2020 |
| HTC carbon hollow spheres | 0.5M H_2_SO_4_ | KOH/SiO_2_ | 760 | 6.4 | Falco et al., 2013 |
| Carbon spheres derived from sodium lignosulfonate | 7M KOH | **—** | 6200 | 5.1 | Pang et al., 2018 |
| Rice straw-derived porous carbon spheres | 6M KOH | KOH | 5000 | 7.46 | Liu et al., 2018 |
| Carbon nano grape skins | 6M KOH | **—** | 51.9 | 9 | Zhang et al., 2017 |
| Activated carbon fibers | 6 M KOH | KOH | 250 | 7.8 | Huang et al., 2016 |
| Activation from waste cottonseed husk | 6 M KOH | KOH | 300 | 10.4 | Chen et al., 2018 |
| GPCSs | 1 M H_2_SO_4_ | K_2_FeO_4_ | 244.5 | 9.10 | This work |
|  |  |  | 4951.7 | 7.29 |  |

**References**

Lin, Hualin, Yeping Liu, Zhexin Chang, Song Yan, Shunchang Liu and Sheng Han 2020. A new method of synthesizing hemicellulose-derived porous activated carbon for high-performance supercapacitors. Microporous and Mesoporous Materials 292. doi: 10.1016/j.micromeso.2019.109707

Chen, H., G. Wang, L. Chen, B. Dai and F. Yu 2018. Three-Dimensional Honeycomb-Like Porous Carbon with Both Interconnected Hierarchical Porosity and Nitrogen Self-Doping from Cotton Seed Husk for Supercapacitor Electrode. Nanomaterials (Basel) 8. doi: 10.3390/nano8060412

Chen, Zhimin, Xiaofeng Wang, Beichen Xue, Wei Li, Zhiyao Ding, Xiaomin Yang, Jieshan Qiu and Zichen Wang 2020. Rice husk-based hierarchical porous carbon for high performance supercapacitors: The structure-performance relationship. Carbon 161: 432-444. doi: 10.1016/j.carbon.2020.01.088

Falco, C., J. M. Sieben, N. Brun, M. Sevilla, T. van der Mauelen, E. Morallon, D. Cazorla-Amoros and M. M. Titirici 2013. Hydrothermal carbons from hemicellulose-derived aqueous hydrolysis products as electrode materials for supercapacitors. ChemSusChem 6: 374-382. doi: 10.1002/cssc.201200817

Pang, Jie, Wenfeng Zhang, Hao Zhang, Jinliang Zhang, Huimin Zhang, Gaoping Cao, Minfang Han and Yusheng Yang 2018. Sustainable nitrogen-containing hierarchical porous carbon spheres derived from sodium lignosulfonate for high-performance supercapacitors. Carbon 132: 280-293. doi: 10.1016/j.carbon.2018.02.077

Liu, Shaobo, Yang Zhao, Baihui Zhang, Hui Xia, Jianfei Zhou, Wenke Xie and Hongjian Li 2018. Nano-micro carbon spheres anchored on porous carbon derived from dual-biomass as high rate performance supercapacitor electrodes. Journal of Power Sources 381: 116-126. doi: 10.1016/j.jpowsour.2018.02.014

Zhang, Guofeng, Jing Zhang, Qing Qin, Yingxue Cui, Wenhao Luo, Yan Sun, Cen Jin and Wenjun Zheng 2017. Tensile force-induced tearing and collapse of ultrathin carbon shells to surface-wrinkled grape skins for high performance supercapacitor electrodes. Journal of Materials Chemistry A 5: 14190-14197. doi: 10.1039/c7ta03113k

Huang, Y., L. Peng, Y. Liu, G. Zhao, J. Y. Chen and G. Yu 2016. Biobased Nano Porous Active Carbon Fibers for High-Performance Supercapacitors. ACS Appl Mater Interfaces 8: 15205-15215. doi: 10.1021/acsami.6b02214
